# Current animal models for the study of congestion in heart failure: an overview

**DOI:** 10.1007/s10741-018-9762-4

**Published:** 2019-01-05

**Authors:** Jirka Cops, Sibren Haesen, Bart De Moor, Wilfried Mullens, Dominique Hansen

**Affiliations:** 1 BIOMED – Biomedical Research Institute, Faculty of Medicine and Life Sciences, Hasselt University, Diepenbeek, Belgium; 2 Doctoral School for Medicine and Life Sciences, Hasselt University, Diepenbeek, Belgium; 3 Hasselt University, BIOMED, Martelarenlaan 42, 3500 Hasselt, Belgium; 4 Department of Nephrology, Jessa Ziekenhuis, Hasselt, Belgium; 5 Department of Cardiology, Ziekenhuis Oost-Limburg, Genk, Belgium; 6 REVAL – Rehabilitation Research Center, Faculty of Rehabilitation Sciences, Hasselt University, Diepenbeek, Belgium; 7 Heart Centre Hasselt, Jessa Ziekenhuis, Hasselt, Belgium

**Keywords:** Animal model, Congestion, Inferior vena cava constriction, Central venous pressure

## Abstract

Congestion (i.e., backward failure) is an important culprit mechanism driving disease progression in heart failure. Nevertheless, congestion remains often underappreciated and clinicians underestimate the importance of congestion on the pathophysiology of decompensation in heart failure. In patients, it is however difficult to study how isolated congestion contributes to organ dysfunction, since heart failure and chronic kidney disease very often coexist in the so-called cardiorenal syndrome. Here, we review the existing relevant and suitable backward heart failure animal models to induce congestion, induced in the left- (i.e., myocardial infarction, rapid ventricular pacing) or right-sided heart (i.e., aorta-caval shunt, mitral valve regurgitation, and monocrotaline), and more specific animal models of congestion, induced by saline infusion or inferior vena cava constriction. Next, we examine critically how representative they are for the clinical situation. After all, a relevant animal model of isolated congestion offers the unique possibility of studying the effects of congestion in heart failure and the cardiorenal syndrome, separately from forward failure (i.e., impaired cardiac output). In this respect, new treatment options can be discovered.

## Introduction

Heart failure is a major public health problem affecting over 23 million people worldwide [1] and is defined as a condition whereby the heart is not able to maintain adequate organ perfusion in the face of normal filling pressures. It comprises both forward failure (i.e., impaired cardiac output) and backward failure (i.e., venous congestion). Venous congestion, as quantified by the central venous pressure (CVP), is the most important factor driving worsening in renal function in patients with heart failure [2]. Congestion is characterized by an increased CVP and intra-abdominal pressure (IAP) (> 8 mmHg) [2, 3]. Hemodynamic congestion is defined as a state of increased intra-cardiac filling without clear clinical manifestations [4]. Clinical congestion occurs later and is evidenced by signs and symptoms such as dyspnea, orthopnea, pulmonary rales, edema, and jugular venous distention [4, 5]. Systemic congestion refers to a general state of congestion characterized by fluid accumulation outside the lungs, accompanied with the aforementioned signs and symptoms [6].

Congestion is classically explained as the consequence of increased cardiac filling pressures, by activation of the neurohumoral and sympathetic nervous system, to compensate a reduced cardiac output (CO). Cardiac filling pressures increase due to ventricular interdependence or due to chronically elevated left-sided filling pressures transmitted back through the pulmonary system into the right ventricle [5], resulting in systemic congestion. The excessive fluid is stored in the splanchnic venous system, which contains highly compliant capacitance veins that can store up to 65% of the total blood volume without repercussions on system hemodynamics [7, 8]. In an attempt to increase the effective circulatory volume, sympathetic stimulation induces vasoconstriction of the splanchnic capacitance vessels and vasodilation of the hepatic veins [9]. The redistribution of this excessive fluid from the capacitance veins to the central venous system, rather than an absolute volume overload, seems to be the most important mechanism contributing to increased cardiac filling pressures [3, 9–11].

The consequences of congestion are not easy to explore in clinical trials, since concomitant forward failure and underlying chronic kidney disease often coexist in patients. Consequently, a wide variety of therapeutics, shown to be beneficial in heart and kidney failure separately, are administered to patients in an attempt to reduce congestion. However, in the clinical setting, the treatment for congestion remains to be optimized. Therefore, further fundamental research in animal models, realistically representing congestion, is necessary. We are the first to review the existing clinically relevant and suitable animal models for the study of congestion, since adequate experimental modeling of the real-life scenario is crucial to examine disease mechanisms and to develop potential therapeutic strategies. In this paper, we will review the existing animal models of heart failure, induced by backward failure, and more specifically congestion.

## Animal models of heart failure: backward failure

Numerous animal models are available to discuss the cardiorenal syndrome, as reviewed by Hewitson et al. [12]. However, these are based on the Ronco classification, which does not focus on the pathophysiological characteristics of the disease [13]. Hemodynamic changes and venous congestion are considered to be the main driving forces contributing to worsening in renal function in heart failure and the cardiorenal syndrome. Heart failure includes both forward failure and backward failure. During backward failure, one or both ventricles fail to eject blood normally thereby causing back pressure on the atria and the venous system and eventually resulting in venous congestion. Eventually, the cardiorenal syndrome develops as renal function is affected by venous congestion [2, 14]. Heart failure associated with congestion is termed congestive heart failure (CHF). Backward failure can be induced in the left- (i.e., myocardial infarction, rapid ventricular pacing) or right-sided heart (i.e., aorta-caval shunt, mitral valve regurgitation, and monocrotaline) in different species by various techniques.

**Fig. 1:**
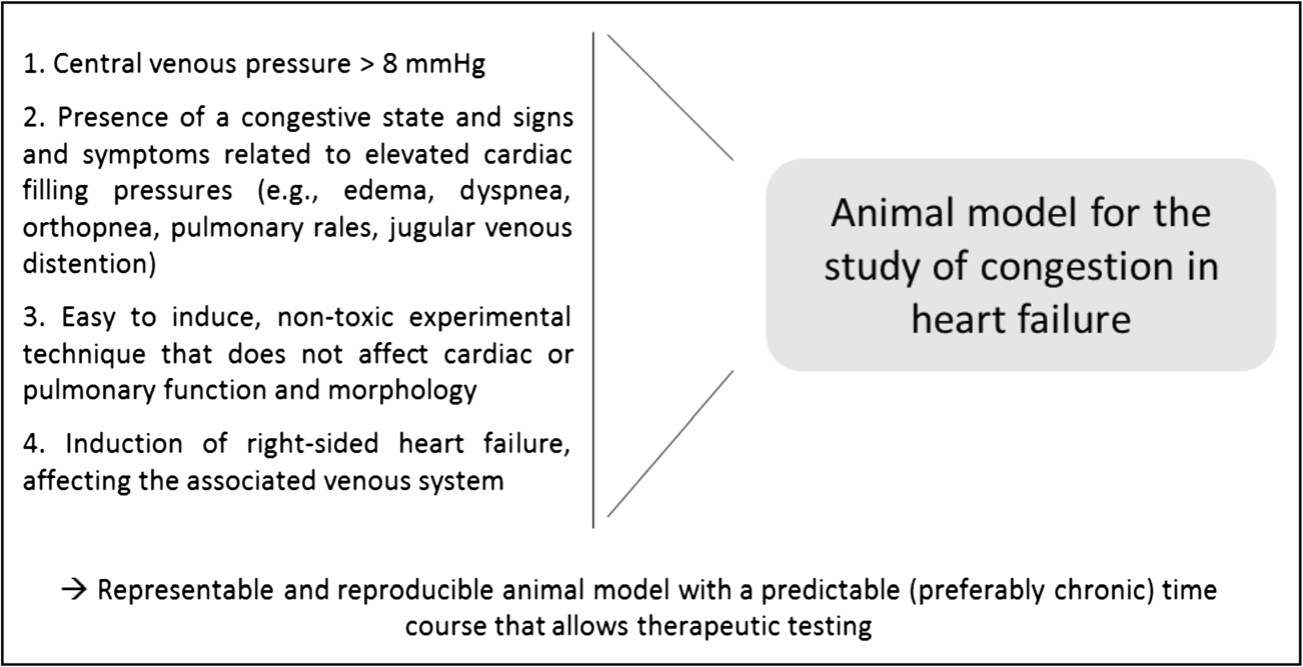
Characteristics of the ideal animal model for the study of congestion in heart failure

These backward failure models only qualify as a relevant model of venous congestion, if the following conditions are met; (1) CVP has to increase above the upper limit of normal (> 8 mmHg), since this is the most important characteristic of congestion contributing to a worsening in renal function in patients; (2) edema or a congestive state is required to be present; (3) the experimental method to induce backward failure should not be toxic or affect left ventricular, valvular or pulmonary function, and morphology; and (4) only the right-sided heart and/or venous system should be affected by backward failure (Fig. 1). The following existing animal models of backward failure do not always meet these requirements as explained in the next sections and Table 1.

**Table 1:** Overview of the existing animal models of heart failure—backward failure

Model	Experimental technique	Technical challenges	Advantages	Disadvantages	Species	References
Myocardial infarction	LAD coronary artery ligation	- Challenging technique - High mortality	- Leading cause of CHF - Widely accepted technique to induce CHF - Congestive features are present	- Varying of degree of myocardial ischemia - Heart failure can be temporary due to compensatory changes - CVP is minimally increased - Left ventricular function/morphology is affected with induction of left-sided heart failure	Rat Rat	Pfeffer et al. 1979 [15] Francis et al. 2001 [16]
Rapid ventricular pacing	External or internal pacemaker to induce arrhythmias	- Choice to pace at a desired bpm	- Simple instrumentation - Neurohumoral activation as in patients - Cessation restores hemodynamics - Congestive features are present	- In patients, CHF develops prior to arrhythmias - CVP is moderately increased - Left ventricular function is affected with induction of left-sided heart failure	Dog Dog Dog Dog Dog	Riegger et al. 1982 [17] Moe et al. 1989 [18] Howard et al. 1988 [19] Ohno et al. 1994 [20] Wilson et al. 1987 [21]
Aorta-caval shunt	Perforation of aorta and inferior vena cava	- Challenging technique	- Congestive features are present - High-output failure and cardiac hypertrophy - Increased CVP	- Decreased MAP - Arterial and venous blood mix - Unpredictable time course - Induction of left- AND right-sided heart failure	Rat Dog Rat Rat Rat Rat	Langenickel et al. 2000 [22] Porter et al. 1983 [23] Flaim et al. 1979 [24] Garcia et al. 1990 [25] Abassi et al. 2011 [26] Liu et al. 1991 [27]
Mitral regurgitation	Chordae disruption by catheter-based method	- Challenging technique	- Minimally invasive - Congestive features are present	- Anatomic changes in mitral valve - Left ventricular function is affected with induction of left-sided heart failure - No clear increase of CVP	Dog Dog Rat Dog	Young et al. 1996 [28] Kleaveland et al. 1988 [29] Ichikawa et al. 1990 [30] Spinale et al. 1993 [31]
Monocrotaline	Varying dose of MCT injection	- The optimal dose to induce pulmonary hypertension without a high mortality	- Induction of pulmonary hypertension and right ventricle failure - Increased CVP	- Toxicity/safety issues - Altered pulmonary function/morphology	Rat Minipig Dog Rat Rat Rat Rat	Werchan et al. 1989 [32] Zeng et al. 2013 [33] Chen et al. 1997 [34] Zhang et al. (2004) [35] Chen et al. (2001) [36] Cui et al. (2009) [37] Angelini et al. 2016 [38]
Pulmonary artery banding	Suture, clip, or inflatable ring around pulmonary artery, proximal to the right ventricle	- Challenging technique - Suture, clip, or inflatable ring, no consensus	- Chronic right ventricular pressure overload and failure - Increased CVP - Effect on kidneys and liver	- Decreased CO	Rat Rat Rat Rat	Schou et al. 2007 [39] Fujimoto et al. 2016 [40] Mendes-Ferreira et al. 2016 [41] Olivetti et al. 1988 [42]
IVC constriction: subdiaphragmatic venous congestion	Saline infusion, surgical wire, metal clamp, adjustable band, or constrictor cuff	- Challenging technique and no consensus about experimental technique	- Increased CVP - Systemic venous pressure overload - Effect on heart, kidneys, and liver	- Variation in anatomical location of IVC constriction	Rat and dog	[43–71]

The experimental technique, technical challenges, advantages and disadvantages, and species specified for each animal model of backward heart failure. The subdiaphragmatic venous congestion animal model is elaborated in detail in Table 2. *LAD* left anterior descending artery, *CHF* congestive heart failure, *bpm* beats per minute, *MAP* mean arterial pressure, *MCT* monocrotaline, *CVP* central venous pressure, *CO* cardiac output, *IVC* inferior vena cava

### Myocardial infarction

Myocardial ischemia and subsequent left ventricular (LV) remodeling is the leading cause of CHF [72]. In rodents, the induction of myocardial infarction (MI), by ligation of the left anterior coronary artery, is a commonly used model of CHF, since congestive features are present due to increased filling pressures (LVEDP 6.1 ± 2.0 in MI rats vs. 4.5 ± 1.2 in control rats, *p* < 0.05) [15, 16, 73]. In addition, Pfeffer et al. [15] demonstrated that right atrial pressure rises (4.4 ± 0.6 mmHg in MI rats vs. 0.4 ± 0.2 in control rats) when the infarction covers more than 46% of total LV endocardial circumference, accompanied by a reduction in systolic (118 ± 3 mmHg in MI rats vs. 43 ± 3 mmHg in control rats, *p* < 0.01) and mean arterial pressure (105 ± 3 mmHg vs. 121 ± 3 mmHg, *p* < 0.01). This eminent sign of hemodynamic congestion is positively correlated with increased blood arginine vasopressin concentrations (*R*^2^ = 0.5949, *p* < 0.05), contributing to sodium and water retention (*p* < 0.05) [16]. However, acute coronary occlusion often fails to induce stable heart failure because compensatory changes, such as neurohormonal activation, development of a collateral circulation, and left ventricular dilation to maintain stroke volume, do occur and animals rarely develop the atherosclerotic lesions associated with heterogeneous blood flow and inconsistent myocardial lesions, typically seen in humans [74]. In addition, the degree of myocardial ischemia resulting from coronary artery occlusion may vary widely, as shown by Lowe et al. [75] in which occlusion of the same vessel at the same anatomical site in dogs resulted in a significant variation in infarct size (< 10% to > 70%). Much of this variation can be explained by differences in the pattern of distribution of the occluded coronary artery [75]. However, due to this large variation, this model is less relevant since repetitions of the animal model should ideally yield the same degree of myocardial ischemia.

### Rapid ventricular pacing

Arrhythmias are often involved in the CHF process as well [76]. Therefore, rapid ventricular pacing-induced tachycardia, often applied in dogs, is used to mimic arrhythmias as seen in patients as it requires simple instrumentation and induces neurohumoral activation closely related to the clinical situation, after 8 weeks of pacing (increase in plasma norepinephrine from 293 ± 15 pg/ml at baseline to 1066 ± 99 pg/ml after 8 weeks of pacing (*p* < 0.01) vs. 270 ± 34 g/ml in control group; increase in plasma renin activity from 1.4 ± 0.4 ng/ml/h at baseline to 10.2 ± 2.4 ng/ml/h after 8 weeks of pacing (*p* < 0.01) vs. 1.7 ± 0.5 ng/ml/h in control group; increase in plasma aldosterone from 124 ± 42 p/ml at baseline to 577 ± 151 pg/ml after 8 weeks of pacing (*p* < 0.01) vs. 124 ± 43 pg/ml in control group) and the neurohumoral activation returns to baseline levels after resumption of sinus rhythm [17, 18]. After 1–2 months of pacing, the rapid ventricular pacing model is characterized by increased LV filling pressures (LVEDP 34.3 ± 7.7 in paced dogs vs. 9.8 ± 4.6 mmHg in control group, *p* < 0.05) [20], pulmonary wedge pressures (26 ± 8 mmHg in paced dogs vs. 10 ± 3 mmHg in control dogs, *p* < 0.01), and right atrial pressures (13 ± 3 mmHg in paced dogs vs. 4 ± 1 mmHg in control dogs, *p* < 0.01), and is associated with ascites, pulmonary congestion, and low-output failure (CO 116 ± 14 ml/min/kg in paced dogs vs. 130 ± 20 ml/min/kg in control group, *p* < 0.01) [20, 21]. Cessation of pacing restores hemodynamic alterations within 4 weeks, which is a unique feature of this CHF model [19]. However, this model fails to demonstrate the true underlying mechanisms of CHF since patients develop CHF first before fatal ventricular arrhythmias occur [77].

### Aorta-caval shunt

Chronic volume overload can be achieved in rats and dogs by the creation of aorta-caval shunt (ACS), also known as an arteriovenous fistula [22, 23]. The application of a shunt results in a decreased mean arterial pressure (89 ± 1 mmHg in ACS rats vs. 108 ± 2 mmHg in control rats) and increased cardiac weight (1496 ± 45 mg in ACS rats vs. 1079 ± 35 mg in control rats, *p* < 0.01), central venous pressure (16.4 ± 3.9 mmHg in ACS rats vs. 4.5 ± 1.6 mmHg in control rats, *p* < 0.05) and left ventricular end-diastolic pressure (8.6 ± 0.9 mmHg in ACS rats vs. 5.7 ± 0.7 mmHg in control rats, *p* < 0.01) [22], thereby leading to ascites, edema, and pleural congestion [24, 26]. To create a shunt or fistula, the abdominal inferior vena cava (IVC) and aorta are exposed by a laparotomy. The aorta is punctured with an 18-gauge needle and the needle is advanced into the vessel, perforating the adjacent wall between the aorta and vena cava until penetration in the vena cava [25]. Usually, a shunt is created between the aorta and vena cava, the femoral artery and femoral vein, or the carotid artery and internal jugular vein. This experimental model is considered a unique model of high-output heart failure, with a concomitant higher CO (114.9 ± 15.5 ml/min in ACS rats vs. 72.6 ml/min in control rats, *p* < 0.05), and cardiac hypertrophy [26, 27]. However, the time course for the development of heart failure is less predictable. In addition, arterial blood is able to mix with venous blood, creating artificially increased cardiac filling and central venous pressures, which is not seen in the clinical situation.

### Mitral valve regurgitation

Volume overload can also be induced in a canine model by mitral valve regurgitation by a catheter-based method of chordae disruption [28]. Chronic mitral regurgitation produces left ventricular dilatation and hypertrophy as evidenced by an increased end-diastolic volume (48 ± 9 ml at baseline vs. 85 ± 19 ml after 3 months, *p* < 0.01), end-systolic volume (19 ± 5 ml at baseline vs. 27 ± 7 ml after 3 months, *p* < 0.05), end-diastolic pressure (9 ± 3 mmHg at baseline vs. 19 ± 6 mmHg after 3 months, *p* < 0.01), stroke volume (29 ± 7 ml at baseline vs. 58 ± 14 ml after 3 months, *p* < 0.01), left ventricular mass (71 ± 13 g at baseline vs. 90 ± 10 g after 3 months, *p* < 0.01), and a decreased CO (2.30 ± 0.61 l/min at baseline vs. 1.80 ± 0.64 l/min after 3 months, *p* < 0.05) and mean aortic pressure (100 ± 11 mmHg at baseline vs. 78 ± 8 mmHg after 3 months, *p* < 0.01) [29]. Additionally, mean pulmonary artery pressure (13 ± 2 mmHg, at baseline vs. 19 ± 5 mmHg after 3 months, *p* < 0.05) was also significantly increased, and hypothetically, this augmented pulmonary artery pressure may also be transmitted back to the venous system [29]. Indeed, Ichikawa et al. (1989) reported that CVP increases in a canine model of mitral valve regurgitation, although not yet reaching statistical significance (6.0 ± 1.8 cmH_2_O (= 4.4 ± 1.3 mmHg) in mitral regurgitation group compared to 4.6 ± 1.4 cmH_2_0 (= 3.4 ± 1.0 mmHg) in control group) [30]. Notwithstanding, CVP of this particular animal model is not commonly reported in literature. Hence, it is not clear if mitral valve regurgitation has a clear effect on CVP. Myocytes are lengthened (94 ± 4 μm in mitral regurgitation dogs vs. 218 ± 8 μm in control group, *p* < 0.05) and demonstrate a reduced contractility [31]. As a result, chronic volume overload is induced, leading to left ventricular dilation and heart failure. This model has the advantage of being minimally invasive despite the fact that anatomic changes in the mitral valve are produced. However, the validity of this animal model is disputable.

### Monocrotaline

Monocrotaline (MCT) injections have been used in rats and minipigs to induce pulmonary hypertension (right ventricular systolic pressure in rats; 77 ± 13 mmHg in MCT vs. 26 ± 2 mmHg in control group, *p* < 0.01 and mean pulmonary artery pressure in minipigs; 24.62 ± 1.38 mmHg in MCT vs. 15.19 ± 0.70 mmHg in control group, *p* < 0.01) and right-sided heart failure [32, 33]. MCT is a pyrrolizidine alkaloid that causes a pulmonary vascular syndrome characterized by proliferative pulmonary vasculitis, pulmonary hypertension, and cor pulmonale [78]. The mechanism causing pulmonary hypertension remains poorly understood [79]. Nonetheless, MCT is known to increase capillary permeability and to induce interstitial edema, fibrosis, macrophage accumulation, and alveolar edema [78]. Eventually, increased pulmonary vascular resistance leads to pressure overload of the right ventricle, as evidenced by an increased right ventricular weight in both rats (0.086 ± 0.007 g in MCT vs. 0.038 ± 0.001 g in control group, 100% increase) and minipigs (11.05 ± 0.5 g in MCT vs. 8.6 ± 0.3 g in control group, *p* < 0.01) subjected to MCT treatment [32, 33]. Depending on the dose, pulmonary hypertension is developed in a few weeks [79]. Chronic pulmonary hypertension is characterized by a significantly increased mean right ventricular pressure (13.2 ± 0.6 mmHg in MCT vs. 9.2 ± 0.3 mmHg in control group, *p* < 0.0001) [34], mean pulmonary artery pressure (24.9 ± 3.7 mmHg in MCT vs. 15.6 ± 1.6 mmHg in control group, *p* < 0.0001) [33], and CVP (5.7 ± 2.8 mmHg in MCT vs. 0.9 ± 0.3 mmHg in control group, *p* < 0.01) [35]. CVP increased on average by 414 to 767% after MCT treatment [35–37]. MCT also contributes to kidney injury as demonstrated by significantly increased serum creatinine (3.06 ± 1.3 pg/ml in MCT rats vs. 0.54 ± 0.23 pg/ml in control group, *p* < 0.05) and serum (562.7 ± 93.34 ng/ml in MCT rats vs. 245.3 ± 58.19 ng/ml in control group, *p* < 0.05), renal and cardiac tissue (70,680 ± 4337 arbitrary units (AU) in MCT rats vs. 32,120 ± 4961 AU in control rats, *p* < 0.01), and neutrophil gelatinase-associated lipocalin (NGAL) levels in rats [38]. However, MCT-related toxicity to the myocardium restricts the relevance of this model to study right ventricular failure [80].

### Pulmonary artery banding

Pulmonary artery banding (PAB) causes chronic pressure overload leading to right-sided heart failure and is performed by placing a suture, clip, or inflatable ring around the pulmonary artery proximally to the right ventricle [80]. Consequently, right ventricular systolic (114.3 ± 7.1 mmHg in PAB rats vs. 36.1 ± 1.7 mmHg in control rats, *p* < 0.05) and diastolic pressures (5.4 ± 1.1 mmHg in PAB rats vs. 3.3 ± 1.1 mmHg in control rats, *p* < 0.05) rise [39], CO is reduced (78.2 ± 27.6 ml/min in PAB rat vs. 150.1 ± 31.2 ml/min in control rats, *p* < 0.01), right ventricular weight increases up to 100–200%, and hepatic function is affected, as evidenced by increased plasma liver enzymes (plasma alkaline phosphatase 160 ± 7 U/L in PAB rats vs. 105 ± 7 U/l in control rats, *p* < 0.05) [39, 40]. PAB is an effective method to induce right-sided heart failure, accompanied by signs of backward failure, such as hepatic congestion (43% of PAB rats), ascites (29% of PAB rats), and hydrothorax (43% of PAB rats) [39, 40]. Hepatic fibrosis develops due to tissue hypoxia resulting from a low CO [40]. Mendes-Ferreira et al. [41] showed that a mild PAB constriction resulted in cardiac hypertrophy with a preserved function, while a severe constriction leads to right ventricle dysfunction, remodeling, and fibrosis in just 3 weeks. Impairment of the right ventricular function increases right ventricular systolic pressure (71 ± 12 mmHg in PAB rats vs. 33 ± 11 mmHg in control rats, *p* < 0.0001) and right ventricular end-diastolic pressure (10 ± 3 mmHg in PAB rats vs. 3 ± 1 mmHg in control rats, *p* < 0.0001) which is transmitted back to the venous system, thereby disturbing venous return and increasing central venous pressure (10 ± 3 mmHg in PAB rats vs. 2 ± 0.2 mmHg in control rats, *p* < 0.0001) [40, 42].

In conclusion, the PAB animal model seems to be the most relevant and appropriate animal model of backward failure to induce congestion, since PAB augments the right ventricular pressure and CVP resulting in a congestive state and right-sided heart failure.

## Animal models of isolated systemic congestion

In the previous section, we summarized the available animal models of heart failure induced by backward failure. None of the models described above fully qualify as a clinically relevant model of congestion. The most commonly employed surrogate for venous congestion has been central venous pressure (CVP). Current knowledge about CVP contributing to renal dysfunction originates from animal experiments performed in the 1850s. Ludwig (1856) demonstrated that urine output decreases as soon as the CVP is raised above 10 mmHg [81]. Later, hemodynamic experiments performed on isolated canine and rat kidneys confirmed the negative impact of venous congestion on renal function, as a CVP > 19 mmHg was transmitted backwards leading to an increased renal interstitial pressure, sodium retention, and a reduced renal blood flow [82, 83]. More recently, the ESCAPE trial showed that right atrial pressure is the only hemodynamic parameter associated with renal insufficiency [84], suggesting an important role for (renal) congestion [85]. Based on these arguments, an animal model with an increased CVP seems to be the most appropriate animal model of congestion and should be preferred.

### Saline infusion

Recently, a rat model of acute renal congestion has been described by Komuro et al. [86]. In this model, the femoral vein was cannulated with an indwelling catheter for the simultaneous monitoring of the CVP and injection of saline to create the acute renal congestion model. A bolus of saline is injected until the CVP increased to 10–15 mmHg [86]. However, being an acute model of renal congestion, the chronic phase cannot be investigated and systemic congestion was not induced. Hence, this model is not preferable.

### Inferior vena cava constriction: subdiaphragmatic venous congestion

In the past, efforts have been made to induce isolated venous congestion in animal models by restricting the diameter of the inferior vena cava (IVC) (Table 2). In this way, the CVP increases above the upper limit of normal (> 8 mmHg), thereby inducing venous congestion in a similar way as seen in patients [2]. Tying a surgical wire [43–58]; inserting a metal clamp [59, 60], adjustable band [61–64], or constrictor cuff around the IVC [65, 66]; or placing an inflatable balloon via the femoral vein [49, 53, 67–69] are frequently used techniques to constrict the IVC. As a result, CVP varied between 11 to 15 mmHg below the constriction while the CVP above the constriction remained unchanged [44, 65].

**Table 2:** Overview of the more specific existing animal models of subdiaphragmatic venous congestion induced by inferior vena cava constriction, proposed to be suitable for translation to the clinical situation

Experimental technique to constrict IVC	Anatomical position	Degree of constriction	Hemodynamics/echocardiography	Species	Time frame	References
Constriction by tying a wire around abdominal IVC and metal rod	Subdiaphragmatic	20% of initial internal cross-sectional area	Hemodynamics	Rat	Chronic	Yates et al. 1958 [43]
Constriction of thoracic IVC	Unknown	1/3 or 2/3 of original diameter	Hemodynamics	Dog	Chronic	Davis et al. 1953 [44]
Constriction of IVC, exact technique unknown	Thoracic and suprarenal	Unknown	Hemodynamics	Dog	Chronic	Seitchik et al. 1960 [45]
Partial constriction of thoracic IVC with umbilical tape	Thoracic	Unknown	Hemodynamics	Dog	Subacute	Levy et al. 1972 [46]
Complete ligation of abdominal IVC; exact technique unknown	Suprarenal and subhepatic	Unknown	–	Rat	Acute	Fitzsimons et al. 1982 [47]
Ligation of abdominal IVC; exact technique unknown	Pre-, inter-, or post-renal	Unknown	–	Rat	Acute	Reinhardt et al. 1951 [48]
Partial obstruction of IVC using an inflatable balloon inserted via the femoral vein/ligation of IVC with cellophane strip	Thoracic	Balloon: unknown Cellophane strip: 50%	–	Dog	Unknown	Crumb et al. 1977 [49]
Ligation by tying a wire around abdominal IVC	Suprarenal	Unknown	–	Rat	Chronic	Du Rietz et al. 1979 [50]
Ligation by tying a wire around abdominal IVC	Supra- and subrenal	Unknown	–	Rat	Acute	Mann et al. [51]
Constriction by tying a wire around abdominal IVC	Between hepatic and renal veins	Unknown	Hemodynamics + echocardiography	Dog	Acute	Schrier et al. 1971 [52]
Partial obstruction of IVC using an inflatable balloon inserted via the femoral vein/placement of a snare around IVC	Thoracic	Unknown	Hemodynamics	Dog	Acute	Anderson et al. 1974 [53]
Constriction by tying a wire around abdominal IVC	Subdiaphragmatic	Unknown	Hemodynamics	Rat	Subacute	Ishikawa et al. 1986 [54]
Constriction by tying a wire around abdominal IVC and polyethylene tube	Subdiaphragmatic	Unknown	–	Rat	Subacute	Kawamura et al. 1986 [55]
Ligation of abdominal IVC;	Subrenal	Unknown	–	Rat	Acute	Zhou et al. 2009 [56]
Constriction of the thoracic IVC by tying a wire round IVC and a steel wire of 0.6 mm	Subdiaphragmatic	70% or original diameter	Hemodynamics	Rat	Chronic	Simonetto et al. 2016 [57]
Ligation by tying a wire around abdominal IVC	Subdiaphragmatic	1/4 of the original diameter	–	Rat	Chronic	Akiyoshi et al. 1999 [58]
Constriction of the thoracic IVC using a metal spiral clamp	Unknown	Clamp with inner diameter clamp of 1.1–1.3 mm	–	Rat	Subacute	Bagrov et al. 1982 [59]
Constriction of thoracic IVC using an adjustable metal clamp	Thoracic	Unknown	Hemodynamics	Dog	Subacute	Lifschitz et al. 1973 [60]
Constriction of thoracic IVC using an adjustable band	Thoracic	Half of the original diameter	Hemodynamics + echocardiography	Dog	Subacute	Lisy et al. 2000 [61]
Constriction of thoracic IVC using an adjustable band	Thoracic	Half of the original diameter	Hemodynamics	Dog	Subacute	Lisy et al. 2005 [62]
Constriction of thoracic IVC using an adjustable band	Thoracic	Half of the original diameter	Hemodynamics	Dog	Subacute	Clavell et al. 1993 [63]
Constriction of thoracic IVC using an adjustable band	Thoracic	Half of the original diameter	Hemodynamics	Dog	Subacute	Wei et al. 1997 [64]
Constriction of the thoracic IVC using an inflatable constrictor cuff	Thoracic	Unknown	Hemodynamics	Dog	Subacute	Paganelli et al. 1988 [65]
Constriction of thoracic IVC using an inflatable constrictor cuff	Unknown	Unknown	Hemodynamics	Dog	Subacute	Watkins et al., 1976 [66]
Partial obstruction of IVC using an inflatable balloon inserted via the femoral vein/partial occlusion of thoracic IVC using umbilical tape	Thoracic	Unknown	Hemodynamics	Dog	Acute/subacute	Auld et al. 1969 [67]
Partial obstruction of IVC using an inflatable balloon inserted via the femoral vein	Unknown	Unknown	Hemodynamics	Dog	Acute	Katz et al. 1959 [68]
Partial obstruction by tying a wire around IVC or by using an inflatable balloon inserted via the femoral vein	Subdiaphragmatic	Unknown	Hemodynamics	Dog	Acute	Cirksena et al. 1966 [69]
Constriction of the thoracic IVC by tying a wire round IVC and a 20G needle	Thoracic IVC, above diaphragm	Against a 20 gauge needle (0.812 mm)	Hemodynamics + echocardiography	Rat	Chronic	Cops et al. 2018 [70] Cops et al. 2018 [71]

The experimental technique, anatomical position, degree of constriction, hemodynamic, or echocardiographic measurements to confirm presence of increased venous pressure, species, and time frame specified for each subdiaphragmatic venous congestion animal model are noted. Time frame is stated as acute (minutes to hours), subacute (days), or chronic (weeks). *IVC* inferior vena cava

IVC constriction affects the kidneys, heart, and liver, thereby inducing subdiaphragmatic venous congestion. Regarding the impact of congestion on the kidneys, subdiaphragmatic venous congestion in dogs [44, 52] and rats [59] is associated with decreased urinary sodium excretion and urine volume, but without significant changes in glomerular filtration rate (GFR). Reinhardt et al. [48] demonstrated that constriction of the IVC above the renal veins resulted in a progressive fall of urinary volume from 72 to 0.4% of total water uptake. In addition, Ishikawa et al. [54] showed that subdiaphragmatic venous congestion reduced water excretion and renal blood flow, without changing GFR. An increased renal venous pressure leads to fluid and sodium redistribution, originally destined for urinary excretion, into the lymphatic system, thereby accounting for the attenuated urine flow and fluid and sodium retention in heart failure [68]. Second, subdiaphragmatic venous congestion may affect the heart by reducing CO and this being the most likely stimulus for the observed sodium retention [52]. Finally, the liver is a vulnerable target of subdiaphragmatic venous congestion since an increased CVP is transmitted back to the hepatic veins [87], which clinically contributes to the development of fibrosis [58] and eventually to congestive hepatopathy, possibly through the mechanism of sinusoidal thrombosis, according to Simonetto et al. [57].

Most of the existing animal models of subdiaphragmatic congestion do not meet the criteria to be a clinically relevant animal model of isolated congestion (Table 2). First, the exact technique to induce congestion [44, 45, 47, 48, 55, 56], the anatomical location of where the constriction is applied [44, 46, 49, 52, 53, 59–68], and the degree of constriction were not always properly described [46, 47, 50, 52, 54, 56, 65, 66, 68, 69]. Second, it was not always clear whether venous congestion was actually induced, since hemodynamic or echocardiographic measurements were not performed in these studies [46–52, 54–56, 58, 59, 69]. Third, often a local and no systemic congestion was induced, when the abdominal IVC was constricted at the level of the renal or hepatic veins [43, 47, 48, 50, 51, 54–58, 68], in contrast to patients in which the abdomen’s entire venous system is congested, potentially explaining contradicting results in animal models versus clinical trials. Fourth, the chronic phase of congestion—weeks to months—was not investigated as the constriction was only maintained for hours to days, or the animals were only often studied for a few days [44–56, 58–69]. Finally, the effect of constriction on abdominal organ function, besides the kidneys, was not investigated [47, 51, 54, 58, 61–64, 67–69]. Taken together, the lack of sufficient information regarding the application of acute or chronic constriction, using a wide variety of techniques on different anatomical positions, makes it difficult to compare results.

## Development of a new rat model of abdominal venous congestion

Recently, the authors developed a new rat model of abdominal venous congestion by addressing these aforementioned limitations [70]. They opted to constrict the IVC in the thoracic cavity in an easy-accessible rat model. In this way, all abdominal organs are affected by the increased CVP and abdominal venous congestion is developed consequently. Briefly, a permanent constriction above the diaphragm was applied by tying a surgical wire around the IVC and a 20-gauge (0.812 mm) needle, after which the needle was immediately removed. The chronic effects of abdominal venous congestion were investigated in this model for a period of 12 weeks. The main findings of this study are that (1) the average abdominal venous pressure of the IVCc group was significantly increased compared to the SHAM group (mean 13.8 mmHg in IVCc rats vs. 4.9 mmHg in SHAM rats, *p* < 0.01); (2) kidney function worsened and renal morphometry was altered in IVCc rats, as indicated by a significantly increased plasma creatinine (median 0.33 mg/dl in IVCc rats vs. 0.28 mg/dl in SHAM rats, *p* < 0.05), plasma cystatin C (median 2.11 mg/dl in IVCc rats vs. 1.25 mg/dl in SHAM rats, *p* < 0.01), urinary albumin (median 86.4 mg/g creatinine in IVCc rats vs. 24.8 mg/g creatinine in SHAM rats, *p* < 0.05), glomerular surface area and with of Bowman’s space (*p* < 0.05); and (3) cardiac function did not differ between both groups, as a result of abdominal venous congestion induced by thoracic IVC constriction. To summarize, Cops et al. [70] were able to develop a rat model to selectively increase the abdominal venous pressure without compromising cardiac function. These findings exclude the effects of a reduced CO on organ functioning in this rat model. In a next study, this rat model was investigated for a maximal follow-up of 21 weeks and it was demonstrated that selective abdominal venous congestion induces retrogradely conducted glomerular hypertension, without a concomitant change in GFR, and hepatic morphological and functional alterations, despite a preserved cardiac function [71].

In conclusion, this rat model offers the unique possibility of studying abdominal venous congestion in heart failure and by extension in the cardiorenal syndrome [70, 71]. This is important since it remains unclear in which manner venous congestion contributes to cardiac and renal dysfunction in patients, and to develop effective therapeutic strategies in the future.

## General conclusion

Congestion, as part of backward failure in heart failure, is an important player in the pathophysiology of heart failure and the cardiorenal syndrome. Nonetheless, the exact disease mechanisms remain to be elucidated and further fundamental research is necessary. In the past, different techniques have been described to induce backward failure in animal models. Unfortunately, these models are not truly a model of congestion, as explained before. Since an increased CVP is such a strong determinant of congestion, a whole spectrum of techniques has been described to constrict the IVC in an attempt to increase the CVP, above the upper limit of normal, and in this way qualifying as model of congestion. To date, the rat model in which selective abdominal venous congestion was induced by a permanent surgical constriction with a 20-gauge needle seems to be a clinically relevant animal model of congestion, since the drawbacks of the previous subdiaphragmatic venous congestion models are corrected in the current model.
